# Opposing effects of impulsivity and mindset on sources of science self-efficacy and STEM interest in adolescents

**DOI:** 10.1371/journal.pone.0201939

**Published:** 2019-08-27

**Authors:** Lisa K. Marriott, Leigh A. Coppola, Suzanne H. Mitchell, Jana L. Bouwma-Gearhart, Zunqiu Chen, Dara Shifrer, Alicia B. Feryn, Jackilen Shannon

**Affiliations:** 1 OHSU-PSU School of Public Health, Oregon Health & Science University, Portland, OR, United States of America; 2 Departments of Behavioral Neuroscience, Psychiatry, and Oregon Institute for Occupational Health Sciences, Oregon Health & Science University, Portland, OR, United States of America; 3 College of Education, Oregon State University, Corvallis, OR, United States of America; 4 Department of Sociology, Portland State University, Portland, OR, United States of America; Medical College of Wisconsin - Central Wisconsin Campus, UNITED STATES

## Abstract

Impulsivity has been linked to academic performance in the context of Attention Deficit Hyperactivity Disorder, though its influence on a wider spectrum of students remains largely unexplored, particularly in the context of STEM learning (i.e. science, technology, engineering, and math). STEM learning was hypothesized to be more challenging for impulsive students, since it requires the practice and repetition of tasks as well as concerted attention to task performance. Impulsivity was assessed in a cross-sectional sample of 2,476 students in grades 6–12. Results show impulsivity affects a larger population of students, not limited to students with learning disabilities. Impulsivity was associated with lower sources of self-efficacy for science (SSSE), interest in most STEM domains (particularly math), and self-reported STEM skills. The large negative effect size observed for impulsivity was opposed by higher mindset, which describes a student’s belief in the importance of effort when learning is difficult. Mindset had a large positive effect size associated with greater SSSE, STEM interest, and STEM skills. When modeled together, results offer that mindset interventions may benefit impulsive students who struggle with STEM. Together, these data suggest important interconnected roles for impulsivity and mindset that can influence secondary students’ STEM trajectories.

## Introduction

Students’ self-beliefs about their abilities in STEM (i.e. science, technology, engineering, and math) directly correlate with persistence in STEM fields [1, 2], even independent of parents’ education or family income [3]. The secondary school period is an important time for shaping students’ self-beliefs in STEM [3, 4] as well as for building STEM interest. While early interest in science is an important predictive factor for students later choosing a STEM-related career [5, 6], it can be over-shadowed by poor academic performance in math and science courses, thereby altering a student’s self-belief in their ability to succeed in science [3]. These self-beliefs are thought to contribute to student attrition from STEM fields [5, 7].

This study explored whether impulsivity may influence students’ self-beliefs in STEM, as Spinella [8] previously reported impulsivity to be negatively associated with academic grades in college-aged students. Impulsivity describes “a predisposition toward rapid, unplanned reactions to internal or external stimuli without regard to the negative consequences of these reactions to the impulsive individuals or to others” [9]. More operationally, impulsivity describes two different behavioral characteristics: (1) an impairment of behavioral inhibition and (2) a pronounced de-valuation of delayed outcomes [10, 11]. Higher levels of impulsivity are associated with various psychopathologies including certain attention-deficit/hyperactivity disorder (ADHD) subtypes, substance use disorder, conduct disorder, and delinquency [12–16]. In contrast, low impulsivity levels have been associated with compulsivity, obsessive compulsive disorder, and some eating disorders [17, 18]. All individuals are expected to fall somewhere along a continuous scale of impulsivity, even when not exhibiting any psychopathology.

Most impulsivity research investigating academic performance focused on the contexts of ADHD [19, 20], risky behaviors [21, 22], and early childhood self-control/regulation [23, 24] leaving the role of impulsivity as an underlying behavioral trait that may shape students’ academic performance largely unexplored [8, 25], particularly in the context of STEM learning. Impulsive students can have trouble staying on task and may be expected to find learning more challenging, as academic effort in many fields, including STEM, involves practice and repetition of tasks as well as concerted attention to task performance. This may be especially true for mathematics, where content builds on prior knowledge and considerable repetitive practice is needed for mastery. Impulsivity may manifest in students as postponing homework or studying, which can contribute to poor academic performance. As students’ self-beliefs in STEM formed during secondary school can be negatively influenced by poor academic performance [3], it is possible that impulsivity may influence these relationships.

Children diagnosed with ADHD can have trouble in school with sustained attention, hyperactivity, and impulsivity, which can negatively affect learning outcomes [26]. Students with ADHD attain lower academic levels than their peers [27], an effect also found for children who are severely inattentive, hyperactive, and impulsive, but lack a formal diagnosis of the disorder [20, 28, 29]. In the United States, the prevalence of these disorders among children and adolescents range from 5.9%-7.1% for ADHD [30], 5–6% for learning disabilities [31], and 0.6–2.2% for autism spectrum disorder [32]. However, sub-clinical levels of impulsivity may also affect students with or without learning disability classifications.

This study assessed impulsivity in a large cross-sectional sample of secondary students to understand whether sub-clinical levels of impulsivity may affect a larger spectrum of students than previously considered. This study was not designed to be causal nor to identify learning disabilities among students, but rather to explore whether students’ impulsivity levels were associated with measures of STEM persistence, such as STEM interest, science self-efficacy, and self-beliefs toward learning.

## Materials and methods

### Participants and settings

This work was overseen by Oregon Health & Science University’s (OHSU) Institutional Review Board (IRB, protocol #3694) who approved this study. A total of six schools were recruited to participate in the current study based on a prior academic relationship with the investigator (L.K.M.) and school sociodemographics. Schools were offered $500 USD for administering two anonymous surveys to their students during the 2014–2015 school year, with all sites accepting. Sites were distributed across three states (Oregon 1 = two rural schools, 6-8th grades; Washington 2 = one suburban school, 6^th^-8^th^ grades; California *3* = three urban schools, one 7^th^-8^th^, one 9-12^th^, and one 7^th^-12th grades) [33]. All sites permitted use of their facilities, managed interaction with students, and oversaw parental opt-out forms that maintained student anonymity to study staff. The study’s IRB protocol permitted each school to select an opt-in or opt-out procedure for parental notification, with all schools selecting an opt-out procedure in this study. Schools managed parental permissions to maintain student anonymity to OHSU study staff. Schools selected which classes would administer surveys to support maximal participation by all interested students. Selected teachers received an informational packet about the study, which included a teacher informational letter, student information sheets, student surveys, a data intake form, and a prepared paragraph to read to their students describing study goals, survey length, and voluntary participation in the anonymous research. Students were then given an information sheet about the study with time to ask questions. Students provided verbal assent to their teacher to participate and surveys included a printed introduction at the top of each survey reiterating procedures being voluntary and anonymous. Completed surveys were returned to the teacher and immediately sealed in a manila envelope. Completed survey packets were returned to the main office to be mailed to the study team (postage pre-paid).

### Assessment procedures

Two paper-based surveys, approximately 30 minutes in length, were administered to students and separated by one month to lessen survey fatigue for students and reduce class interruption time. To maintain anonymity while permitting linkage of the two surveys, students were asked a series of questions on each survey to generate a unique identification number including a) first two letters of mother’s first name (ID_a), b) day of birth (ID_b), c) last two digits of phone number (ID_c), and d) birth order (ID_d, in the case of twins, etc.). These responses, along with grade, gender, age, and teacher administering the survey, comprised the students’ “unique ID” and was used to match the two surveys using a deterministic matching procedure described below.

### Instruments

Instruments included in Survey 1 included impulsivity, mindset, sources of science self-efficacy, and STEM skills. Instruments included in Survey 2 assessed interest in STEM domains, interest in a STEM career, and questions about learning behaviors.

- Barratt Impulsiveness Scale–short form (BIS-15)–The BIS-15 [34] comprised 15 items measured on a 4-point Likert scale (1–4, with six items reverse scored as previously reported [34, 35]. Higher scores on this scale denote more impulsivity. Subscales (Attentional [A], Motor [M], and Non-Planning [NP]) previously produced Cronbach’s alpha coefficients (α) between α = .60-.78 in university students. In the current study, a total of 2080 students completed all 15 items (α = .75), calculated from its three subscales, A (α = .74, n = 2289), M (α = .61, n = 2282), and NP (α = .68, n = 2273).
- Sources of Science Self-Efficacy–SSSE applied Usher and Parajes’ validated mathematics scale [36] reworded for science [37]. The instrument comprised 24 items that addressed four constructs: mastery experiences (ME), vicarious experiences (VE), social persuasion (P), and psychological and affective state (PH). Items were scored based on a 6-point Likert scale (0–5, scores from 0–120), with higher scores denoting more SSSE. Previous test reliability among 1225 middle and high school students produced α = .87, .71, 85, and .86 for the four constructs, respectively. In the current study, a total of 1899 students completed all 24 items (α = .86), representing a composite measure of SSSE calculated from ME (α = .88, n = 2210), VE (α = .89, n = 2145), P (α = .91, n = 2086), and PH (α = .92, n = 2088).
- Mindset–Mindset describes a student’s felt beliefs of being able to increase personal intelligence, with high values representing the belief that intelligence can be increased through effort (termed “growth mindset”) and that low intelligence is a static trait conferred at birth (“fixed mindset”, [38, 39]. A 20 item instrument designed by Dweck [38, 39] was scored on a 4-point Likert scale (1–4, with 10 items reverse-scored). Items stem from the Theory of Intelligence scale [38], Effort Belief Scale [40], and Patterns of Adaptive Learning Survey [41]. Higher scores on this scale are associated with “growth” mindset whereas lower scores are associated with “fixed” mindset. Current analyses of 1759 students completing all 20 items produced α = .75.
- STEM Skills–Four questions were developed to assess self-reported skills related to using and interpreting data. Each question offered the stem “I am good at projects involving…” with responses of 1) “using a website”; 2) “using data”; 3) “creating graphs”; and 4) “interpreting graphs”. Responses were scored on 5-point Likert scale ranging from Strongly Disagree to Strongly Agree, with higher scores denoting more perceived STEM skills. The current analyses of 2405 students completing the 4 items produced α = .76.
- STEM Interest–A 25-item STEM Semantics survey assessed student perceptions and interest across five STEM domains: 1) science, 2) math, 3) engineering, 4) technology, and 5) a STEM career [42, 43]. Each domain included five questions that used adjective pairs to bookend a 7-point Likert scale, with a subset of items reverse scored. Domain scores were summed for each five question set with higher scores denoting greater STEM interest. A composite STEM interest score was summed from all five subscales. Previous reliability among 174 students ranged from α = .84-.93, with 1575 students completing all 25 items in the current study (α = .93). The five subscales included science interest (α = .89, n = 1807), math interest (α = .90, n = 1812), engineering interest (α = .90, n = 1755), technology interest (α = .90, n = 1784), and interest in a STEM career (α = .92, n = 1785).
- STEM Learning–Four questions from the Index of Learning Styles [44] were used to triangulate findings, as they dichotomize students’ processes for solving mathematics problems and overall learning pace in the context of impulsivity. Selected questions were: 1) “When I am doing long calculations: a) I tend to repeat all my steps and check my work carefully, or b) I find checking my work tiresome and I have to force myself to do it”; 2) “When I solve math problems: a) I usually work my way to the solutions one step at a time, or b) I often just see the solutions but then have to struggle to figure out the steps to get to them”; 3) “I learn: a) at a fairly regular pace. If I study hard, I’ll “get it”, or b) in fits and starts. I’ll be totally confused and then suddenly it all “clicks””; and 4) “In a study group working on difficult material, I am more likely to a) jump in and contribute ideas, or b) sit back and listen”.

### Survey processing and statistical analyses

Paper surveys were scanned using Remark software that populated survey data into Excel. Files were transferred into SAS (9.4) for survey matching with data statistically analyzed using SPSS (IBM, version 24). Statistical modeling was implemented using R (version 3.5.1). Use of multiple statistical software stems from the dataset being independently analyzed by three researchers (ZC, LKM, AF). Geographical location and school demographics were obtained from 2013–2014 NCES data [33].

#### Survey linking procedure

A deterministic matching procedure was used to first match all nine variables (school, gender, grade, ID_a, ID_b, ID_c, age, teacher, and ID_d) with matched records moved to a new dataset. The procedure was repeated down to five variables, with handwriting samples confirming matching at each level (n = 31 total; 100% agreement). This procedure was used to link Survey 1 (n = 2476) with Survey 2 (n = 2115), representing a conservative match rate of 41.4% (n = 875) of anonymous students. Analyses were conducted on all completed items; therefore, comparisons within a survey had larger sample sizes than between surveys.

#### Statistical analyses

Likert scale responses were converted numerically and summed for each subscale and composite total score, with data analyzed as continuous variables (e.g., impulsivity, mindset, SSSE, STEM interest). Cases with missing values were set to missing on the final scale; students with missing scales were not included in calculations.

Linear models were used to understand the impact of STEM interest, impulsivity, and mindset on SSSE after adjusting for gender, grade, and school. There was limited URM data across all schools to adjust for URM. Hierarchical linear mixed models (HLMMs) were considered using interest in STEM domains as independent continuous variables with the addition of grade and gender as covariates and school as a random intercept to account for clustering of students within schools. However, the data could not support HLMMs, and when it could, the intraclass correlation (ICC) was nearly zero, indicating that the random intercept was unnecessary for this particular dataset.

A HLMM on SSSE using impulsivity and mindset as independent variables with school as a random intercept adjusted for grade, gender, and underrepresented minority exemplifies a case where the data supported the model, but the ICC was nearly zero. The model was then re-run as a linear model where school was included as a fixed effect to verify consistency between the two models, which concluded that accounting for clustering at schools is unneeded. Additionally, the linear model was rerun without inclusion of URM as a covariate for comparability with other STEM interest models that lack URM as a covariate. HLMMs were implemented in R using the “lmer” function within the 1.1-18-1 version of the “lme4” package. Linear models were performed using “lm” in R. Cases with any missing values were excluded from the analysis of the HLMM and linear models.

The relationship between SSSE, mindset, impulsivity, and math interest on learning behaviors (dichotomous variables) adjusted for gender, grade, and school were assessed with logistic regression models. SSSE, mindset, impulsivity, and math score were included in the model as quartiles. Similar to previous models, the ICC was nearly zero, affirming the exclusion of a random intercept. Predicated probabilities of SSSE, mindset, and impulsivity were calculated by quartile to discern differences in learning behaviors between students at opposing ends of the scales (e.g., high/low impulsivity, mindset, SSSE, math interest). Logistic regression models were performed using “glm” in R.

#### Data visualization

The statistical models above are robust because they include important covariates of interest in analyses (e.g., gender, grade, school, URM). However, to support visualization of results identified in these models, group means are presented in summary tables along with results from simple statistical tests, such as independent sample t tests (e.g., gender) and ANOVAs with Bonferroni post-hoc tests (e.g., grade). These summary tables are intended to help visualize the magnitude of effects observed in the models, with effect sizes across groups reported using partial eta squared (i.e., partial η^2^, η^2^p) based on established benchmarks defining small (partial η^2^ = 0.01), medium (partial η^2^ = 0.06), and large (partial η^2^ = 0.14) effects [45, 46].

To illustrate the relationship with classroom behaviors that could impact student learning, mean scores for impulsivity, mindset, SSSE, and math interest scores (continuous variables) were reported for each dichotomous answer option selected by students. Results were also graphed to show the proportion of students selecting each answer option across student quartiles for that scale. Graphs reflect mean±SEM, or percentages for student behaviors.

#### Missing data procedures

To control for missing data, since impulsive students may be more likely to skip questions or scales, survey responses were analyzed by student demographics (gender and grade) within and between survey time points. Instrument scores were compared by completion status and demographics to understand if scores differed for students who completed all scales versus a subset of scales.

## Results

### Participants

**Fig 1:**
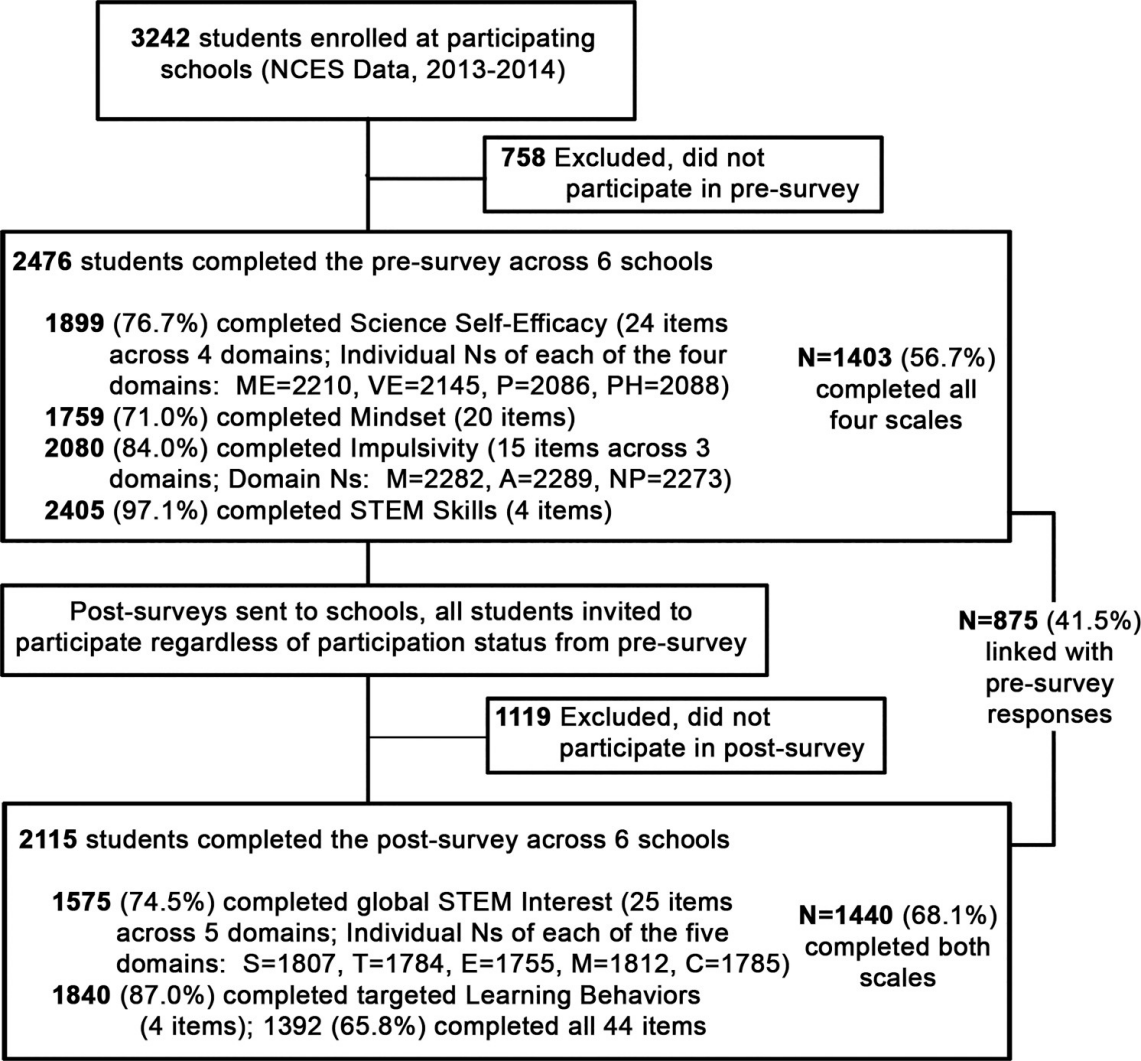
Inclusion criteria and sample sizes for analyses.

A total of 3234 students were enrolled across the six sites (NCES 2015) and had the opportunity to complete survey measures, with 2476 completing Survey 1 and 2115 completing Survey 2. Fig 1 describes inclusion criteria and instrument sample sizes for analyses across the two survey time points. Of the 2476 students in grades 6–12 completed Survey 1, 85.8% were middle school students in U.S. grades 6–8 (Table 1). Participants were 47% female, consistent with NCES data for these participating schools (47.7% female; 58.7% qualify for free or reduced lunch). Racial/ethnic demographics of students were not collected in this study, though NCES data describe that 33.4% qualified as underrepresented minorities (URM) in STEM [47], denoting students who identified as African American (9.5%), Hispanic or Latino (22.6%), or Native American/Alaskan Native (1.3%). Students identifying as “Two or More Races” represent an additional 6.8% of the student population. Survey 2 was completed by a similar number of students, with chi square showing similar distributions in gender (p = .66) but not grade (p<0.001), as less 7^th^ grade students and more high school students participated in Survey 2 (Table 1). Sample sizes and means for each scale are reported in Table 2 along with any subscale differences observed between groups.

**Table 1:** Participant demographics reported by the school and by students completing each survey.

	Overall School Demographics NCES Data (n = 3234)	Survey 1 (n = 2476) *Mindset*, *Impulsivity*, *Science Self-Efficacy*, *STEM Skills*	Survey 2 (n = 2115) *STEM Interest*, *Learning Behaviors*
**Gender**			
Female	1543 (47.7%)	1132 (46.9%)	972 (46.8%)
Male	1691 (52.3%)	1281 (53.1%)	1103 (53.2%)
		n = 2413	N = 2075
**Grade**			
6	493 (15.2%)	449 (18.6%)	422 (20.4%)
7	1089 (33.7%)	1011 (42.0%)	658 (31.9%)
8	1109 (34.3%)	607 (25.2%)	597 (28.9%)
9	157 (4.9%)	136 (5.6%)	132 (6.4%)
10	153 (4.7%)	87 (3.6%)	116 (5.6%)
11	106 (3.3%)	92 (3.8%)	83 (4.0%)
12	127 (3.9%)	26 (1.1%)	56 (2.7%)
		n = 2408	n = 2064

**Table 2:** Means and effect sizes of impulsivity, mindset, sources of science self-efficacy (SSSE), and STEM domain interest across gender and grade.

	Impulsivity	Mindset	Sources of Science Self-Efficacy (SSSE)	Science Interest	Math Interest	Engineering Interest	Technology Interest	Interest in a STEM Career	Interest in STEM Domains (Cumulative Score)
Overall Mean ± SD (n)	33.2, 6.7, n = 2080	60.0, 7.3, n = 1759	67.4, 22.6, n = 1899	24.2, 7.9 (n = 1807)	21.8, 8.8 (n = 1812)	22.6, 8.8 (n = 1755)	25.3, 8.2 (n = 1784)	24.0, 8.5 (n = 1786)	94.1, 24.2 (n = 1575)
**Gender**	*t*(2028) = 1.20, p = 0.23 partial η2 = 0.001	*t*(1723) = -1.99, p<0.05 partial η^2^ = 0.002	t(1856) = 2.69 p = 0.007 partial η2 = 0.004	*t*(1773) = 2.76, p<0.007 partial η2 = 0.004	*t*(1781) = 2.37, p<0.02 partial η2 = 0.003	*t*(1727) = 12.05, p<0.001 partial η2 = 0.078	*t*(1754) = 8.88, p<0.001 partial η2 = 0.043	*t*(1756) = 5.8, p<0.001 partial η2 = 0.019	*t*(1773) = 2.76, p<0.007 partial η2 = 0.049
Male	33.3, 6.4, n = 1090	59.7, 7.5, n = 896	68.8, 21.9, n = 967	24.7, 7.8, n = 939	22.3, 8.6, n = 945	24.9, 8.4, n = 918	26.9, 8.0, n = 928	25.2, 8.4, n = 926	99.1, 23.7, n = 825
Female	32.9, 6.8, n = 940	60.4, 7.0, n = 829	66.6, 23.3, n = 891	23.7, 8.0, n = 836	21.26, 9.0, n = 838	20.0, 8.6, n = 811	23.5, 8.0, n = 828	22.8, 8.5, n = 832	88.3, 23.6, n = 726
**Grade**	*F*(6, 2022) = 3.32, p<0.004 partial η^2^ = 0.01	*F*(6, 1719) = 2.47, p<0.03 partial η^2^ = 0.009	*F*(6, 1850) = 7.31, p<0.001 partial η2 = 0.023	*F*(6, 2148) = 11.56, p<0.001 partial η2 = 0.022	*F*(6, 2086) = 10.36, p<0.001 partial η2 = 0.029	*F*(6, 2040) = 3.76, p<0.001 partial η2 = 0.018	*F*(6, 2034) = 4.24, p<0.001 partial η2 = 0.019	*F*(6, 1863) = 7.93, p<0.001 partial η2 = 0.038	*F*(6, 2148) = 11.56, p<0.001 partial η2 = 0.031
6	32.0, 6.6, n = 371 ^b^	61.3, 6.8, n = 218 ^c^	71.3, 23.1, n = 268 ^a^	25.5, 7.5, n = 352 ^a^	24.1, 8.8, n = 355 ^a^	23.4, 8.3, n = 329 ^a^	26.5, 7.9, n = 341 ^a^	26.4, 7.8, n = 347 ^a^	98.4, 23.0, n = 303 ^a^
7	33.1, 6.3, n = 847	59.9, 7.2, n = 771	66.4, 21.7, n = 797 ^bnz^	24.1, 7.7, n = 552 ^a^	21.8, 8.5, n = 556 ^y^	23.2, 9.0, n = 543 ^a^	25.9, 8.2, n = 542 ^bz^	24.3, 8.5, n = 547 ^bz^	95.5, 23.2, n = 479 ^a^
8	33.3, 7.0, n = 514	60, 7.4, n = 464	70.4, 22.5, n = 498 ^a^	24.4, 8.1, n = 516 ^a^	21.8, 8.5, n = 517 ^y^	22.5, 8.8, n = 505 ^b^	24.7, 8.4, n = 519	24.1, 8.2, n = 513 ^bx^	93.9, 24.5, n = 448 ^a^
9	34.6, 6.8, n = 115 ^y^	58.5, 7.1, n = 111 ^z^	58.3, 24.0, n = 119 ^mx^	20.4, 8.3, n = 117	20.3, 8.9, n = 118 ^x^	19.2, 9.1, n = 117	22.7, 8.0, n = 116	21.0, 8.7, n = 115 ^x^	82.8, 25.0, n = 106
10	34.3, 6.1, n = 77	59, 7.2, n = 68	64.0, 25.0, n = 71	24.9, 7.3, n = 101 ^a^	20.2, 9.2, n = 98 ^x^	23.5, 8.6, n = 100 ^b^	26.7, 7.5, n = 99 ^b^	23.5, 9.5, n = 102 ^c^	95.9, 25.1, n = 91 ^b^
11	33.6, 7.2, n = 83	60.5, 8, n = 76	66.1, 23.9, n = 81	23.5, 7.7, n = 72	18.2, 8.8, n = 72 ^x^	21.1, 7.8, n = 66	24.4, 7.3, n = 73	20.8, 7.7, n = 72 ^x^	87.1, 23.0, n = 60 ^z^
12	34.1, 6.1, n = 22	58.4, 7.3, n = 18	57.9, 17.0, n = 23	23.5, 8.8, n = 51	18.4, 9.4, n = 51 ^x^	20.0, 8.7, n = 52	22.9, 8.2, n = 50	19.6, 9.2, n = 51 ^x^	84.5, 25.5, n = 48 ^y^

Results shown as Mean, SD, and sample size when analyzed by independent sample t-tests (gender) or ANOVA (grade). Higher scores denote more impulsivity, mindset (e.g., “growth” mindset), SSSE, and STEM interest. Effect size benchmarks define small (partial η^2^ = 0.01), medium (partial η^2^ = 0.06), and large (partial η^2^ = 0.14) effects [45, 46]. Bonferroni post-hoc tests were used to determine differences between groups through multiple comparisons. For grade, ^a^ denotes differences between 9^th^ grade students at the p<0.001, ^b^ p<0.01, and ^c^ p<0.05 levels whereas ^x^ denotes differences between 6^th^ grade students at the p<0.001, ^y^ p<0.01, and ^z^ p<0.01 levels. No differences in impulsivity subscales were observed for gender though grade had a small effect on overall impulsivity (p<0.005; partial η^2^ = 0.01), with similar effects observed for both M (p<0.001, partial η^2^ = 0.013) and A (p<0.005; partial η^2^ = 0.016) subscales. Specifically, 9^th^ graders had highest impulsivity as well as motor and attentional subscale scores, though differences were only significant when compared to 6^th^ grade students (p<0.05). For SSSE, only the physiological state (PH) subscale differed between genders (p<0.001; partial η^2^ = 0.014), with males having higher sub-scores than females (male mean = 22.3, SD = 7.4, n = 1081; female mean = 20.5, SD = 8.0, n = 961; *t*(1809) = 5.19, p<0.001). As PH items are reverse-scored, lower numbers denote a higher physiological response. Grade had a small effect on SSSE (p<0.001, partial η^2^ = 0.023) with Bonferroni post-hoc tests showing lower SSSE and ME sub-scores among 9^th^ grade students compared to students in 6-8^th^ grade (p<0.002).

### Interest in STEM domains and career interest are associated with increased sources of science self-efficacy

A significant relationship was observed between interest in all STEM domains (e.g., science, technology, engineering, and math interest) and SSSE when examined by linear models, which included grade, gender, and school in the model (Table 3). The average science self-efficacy score for a male student in 6th grade with average science interest and attending the first school was 68.66 (95% CI: 60.54–76.79). Every unit increase in science interest was associated with a 1.41 average increase in SSSE (p<0.001) after adjusting for other differences. Interest in other STEM domains also related significantly to SSSE, with estimates ranging from 0.84 (STEM career interest) to 0.42 (STEM domain interest). On average, females had significantly lower SSSE than males when adjusted for science interest (-3.35; p<0.05) and math interest (-4.43; p<0.02). School had no significant effect in any of the models except for cumulative STEM domain interest, where one school (School 6) was on average, 9.92 units higher in SSSE than School 1 (95% CI: 0.69–19.16, p<0.04).

**Table 3:** Interest in STEM domains was associated with higher sources of science self-efficacy (SSSE) scores among students in grades 6–12, even after adjusting for gender, grade, and school. STEM domain interest is ranked by impact on SSSE scores.

STEM Domain	Parameters	Estimate	Standard Error	t	Signifcance (p)	95% Confidence Interval
**Science Interest**	Intercept	68.66	4.14	16.6	p<0.001	60.54–76.79
	Science Interest	1.41	0.11	13.11	p<0.001	1.20–1.62
	Grade	-0.18	0.97	-0.19	p = 0.85	1.71–0.85
	Gender	-3.35	1.68	-2.00	p<0.05	-0.06–0.05
**STEM Career Interest**	Intercept	66.72	4.52	14.76	p<0.001	57.84–75.60
	STEM Career Interest	0.84	0.11	7.70	p<0.001	0.62–1.05
	Grade	0.18	1.04	0.17	p = 0.87	-1.87–2.22
	Gender	-2.97	1.82	-1.63	p = 0.10	-6.55–0.61
**Math Interest**	Intercept	66.94	4.56	14.70	p<0.001	57.99–75.89
	Math Interest	0.66	0.11	6.21	p<0.001	0.45–0.87
	Grade	0.49	1.07	0.46	p = 0.64	-1.60–2.59
	Gender	-4.43	1.84	-2.41	p<0.02	-8.04 - -0.82
**Engineering Interest**	Intercept	66.14	4.69	14.11	p<0.001	56.93–75.35
	Engineering Interest	0.643	0.11	5.73	p<0.001	0.42–0.86
	Grade	0.25	1.09	0.23	p = 0.82	-1.88–2.39
	Gender	-2.51	1.94	-1.29	p = 0.20	-6.33–1.31
**Technology Interest**	Intercept	67.64	4.72	14.34	p<0.001	58.38–76.90
	Technology Interest	0.54	0.12	4.51	p<0.001	0.31–0.77
	Grade	0.23	1.09	0.21	p = 0.83	-1.91–2.37
	Gender	-3.68	1.92	-1.92	p = 0.06	-7.44–0.08
**STEM Domain Interest**	Intercept	64.65	4.64	13.94	p<0.001	55.54–73.75
	STEM Domain Interest	0.42	0.04	10.57	p<0.001	0.34–0.50
	Grade	0.89	1.07	0.83	p = 0.41	-1.21–2.98
	Gender	-1.88	1.88	-1.00	p = 0.32	1.82–0.32

Linear models were implemented on SSSE using interest in each STEM domain as independent continuous variables with the addition of grade and gender as covariates and school as a fixed effect. Variables were coded as follows: Gender (Male = 0, Female = 1); Grade (6–12); and School (1–6). Baseline variables for the model were established using grade 6, male gender, and average STEM interest score for that domain. The average SSSE score for 6th grade male students with average STEM interest (e.g. for science interest) was 68.66. Every unit increase in science interest was associated with a 1.41 unit increase in SSSE (p<0.001) while other variables were held constant (i.e., gender, grade, school). The estimates for school are not shown in this table due to space constraints but had no significant effect on any models with the exception of cumulative STEM domain interest where one school (School 6) was 9.92 units higher in SSSE than School 1 (95% CI: 0.69–19.16, p<0.04).

### Impulsivity and mindset have opposing effects on sources of science self-efficacy

Linear models were implemented to understand if school, gender and grade contributed to the relationship between impulsivity and mindset on SSSE, with and without URM included in the model as covariates. In the models, variables were first re-coded to set a baseline (baseline denotes 6th grade, male gender, non-underrepresented minority background [if included in model], and average impulsivity and mindset). The model’s intercept with the inclusion of URM was estimated to be 76.76, with each unit of impulsivity decreasing SSSE by 1.35 units (95% CI: -1.80 to -0.89) and each unit of mindset increasing SSSE by 1.24 units (95% CI: 0.81 to 1.67; **Table 4**). When URM was removed from the model, the model’s intercept was estimated to be 70.84, with each unit of impulsivity decreasing SSSE by 1.28 units (95% CI: -1.44 to -1.11) and each unit of mindset increasing SSSE by 0.96 (95% CI: 0.81 to -1.10). Both impulsivity and mindset significantly contributed to the models, and in turn, the prediction of an individual’s SSSE even after adjusting for grade, gender, and underrepresented minority background (all p<0.001, **Table 4**). In the non-URM model only, SSSE was 4.43 units lower on average for females relative to males (95% CI -6.47 - -2.38, p<0.001). School had a significant impact on the SSSE for two of the six schools (non-URM model), where School 4 was 5.40 units less in SSSE than School 1 (95% CI -10.34 to -0.45, p<0.04) and School 5 was 6.70 units less in SSSE than School 1 (95% CI -10.63 to -2.77, p<0.001).

**Table 4:** Linear models describing the effects of impulsivity and mindset on sources of science self-efficacy, science interest, and math interest after adjusting for gender, grade, and school. Impulsivity was negatively associated with student interest in science and math, as well as with their beliefs in their science abilities (SSSE). Higher mindset scores (“growth” mindset) were positively associated with science and math interest, as well as SSSE.

Outcome	Parameters	Estimate	Standard Error	T	Sig (p)	95% Confidence Interval
**Sources of Science Self Efficacy** (with URM)	Intercept	76.76	4.89	15.72	p<0.001	67.13–86.39
	Impulsivity	-1.35	0.23	-5.85	p<0.0001	-1.80- -0.89
	Mindset	1.24	0.22	5.67	p<0.0001	0.81–1.67
	Grade	-0.51	1.28	-0.40	p = 0.69	-3.03–2.01
	Gender	-5.38	2.74	-1.96	p = 0.05	-10.79–0.031
	Underrepresented Minority	-3.09	3.00	-1.03	p = 0.30	-9.00–2.82
**Sources of Science Self Efficacy** (without URM)	Intercept	70.84	2.08	34.02	p<0.001	66.75–74.92
	Impulsivity	-1.28	0.08	-15.43	p<0.001	-1.44 - -1.11
	Mindset	0.96	0.08	12.73	p<0.001	0.81–1.10
	Grade	0.10	0.64	0.16	p = 0.87	-1.14–1.35
	Gender	-4.43	1.04	-4.25	p<0.001	-6.47 - -2.38
**Math Interest**	Intercept	25.45	1.91	13.32	p<0.001	21.69–29.2
	Impulsivity	-0.37	0.06	-5.93	p<0.001	-0.49 - -0.25
	Mindset	0.04	0.06	0.66	p = 0.51	-0.08–0.16
	Grade	-1.22	0.45	-2.71	p<0.01	-2.11- -0.34
	Gender	-0.80	0.77	-1.03	p = 0.30	-2.31–0.72
**Science Interest**	Intercept	23.46	1.77	13.24	p<0.001	19.98–26.94
	Impulsivity	-0.22	0.06	-3.80	p<0.001	-0.34 - -0.11
	Mindset	0.15	0.05	2.80	p<0.01	0.05–0.26
	Grade	0.25	0.42	0.59	p = 0.55	-0.59–1.07
	Gender	-1.17	0.72	-1.64	p = 0.10	-2.58–0.24

A linear model was implemented on SSSE using impulsivity and mindset as independent continuous variables with the addition of grade, gender, school, and underrepresented minority as covariates. Variables were coded as follows: Gender (Male = 0, Female = 1); URM (URM = 1; Not URM = 0), Grade (6–12); and School (1–6). Baseline variables for the model were established using grade 6, male gender, not underrepresented race/ethnicity, and average impulsivity and mindset scores. The average SSSE score for 6th grade students with average impulsivity and average mindset who are male and are not underrepresented is 76.76 (i.e. baseline SSSE). Every unit increase in impulsivity was associated with a 1.35 unit decrease in SSSE (p<0.001) while other variables were held constant (i.e., gender, grade, URM). In contrast, every unit increase in mindset was associated with a 1.24 unit increase in SSSE (p<0.001). The model was replicated without URM as a covariate to support comparisons with other measures (e.g., science interest, math interest). The estimates for school are not shown in this table due to space constraints but significant effects on SSSE compared to School 1 were observed for two schools (School 4: -5.40, p<0.04 and School 5: -6.70, p<0.001). No school effects were observed for math or science interest models. Female gender was associated with a lower SSSE than male gender (non-URM model; -4.43; <0.001). Grade significantly impacted math interest where each increase in grade level was associated with a 1.22 unit decrease in math interest (p<0.001).

**Fig 2:**
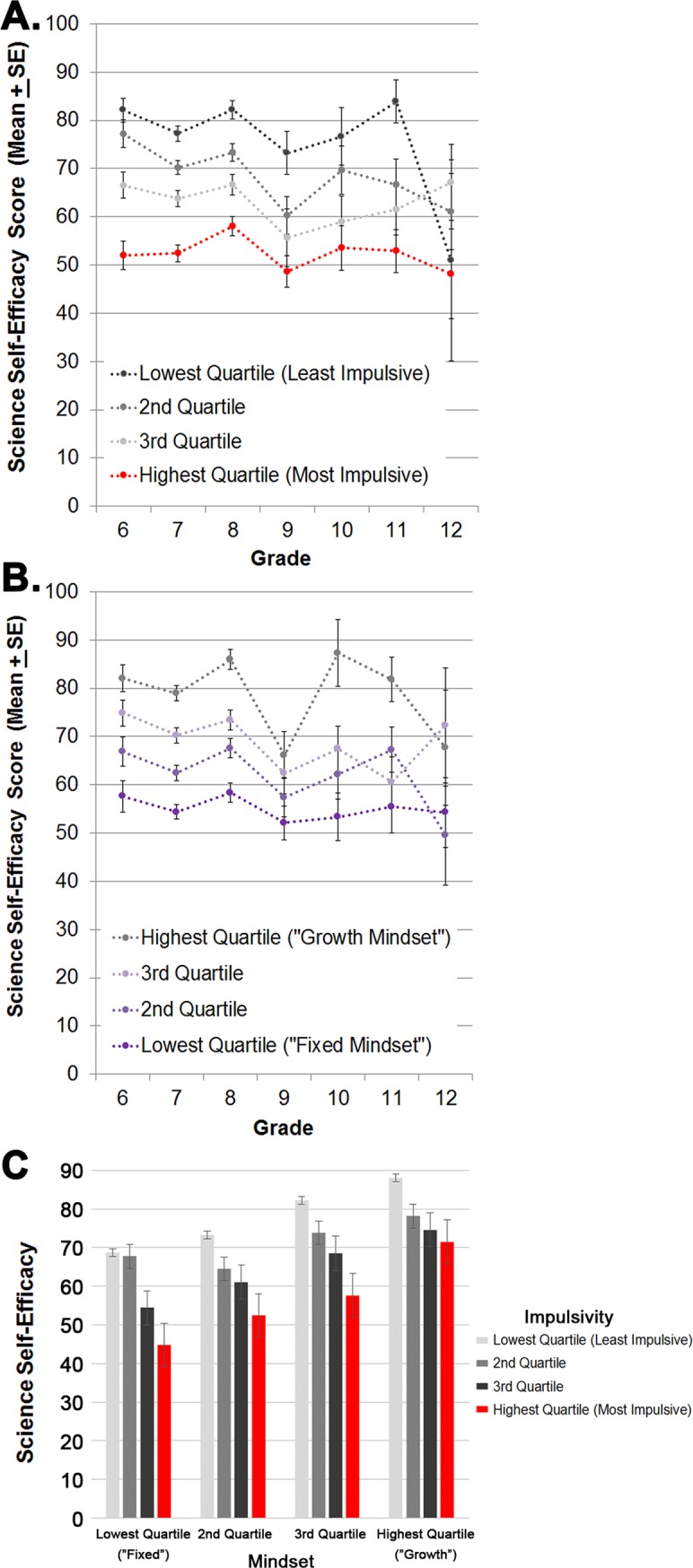
Sources of science self-efficacy (SSSE) scores were influenced by impulsivity and mindset. Impulsivity (A; large negative effect size; p<0.001; partial η^2^ = 0.206) and mindset (B; large positive effect size, p<0.001; partial η^2^ = 0.206). When modeled together (C), higher mindset opposed impulsivity’s negative on SSSE (both p<0.001, no interaction [p = 0.705]). Students with most impulsivity (red bars) yet highest mindset (“growth” mindset) had equivalent science self-efficacy scores to students with least impulsivity and lowest mindset (“fixed” mindset). Every unit increase in impulsivity was associated with a 1.35 unit decrease in SSSE while other variables were held constant (i.e., gender, grade, URM; p<0.0001). In contrast, every unit increase in mindset was associated with a 1.24 unit increase in SSSE. Continuous data were analyzed, with visualization of results shown using quartiles.

Together, impulsivity had a negative relationship with SSSE whereas higher mindset has a positive relationship with SSSE, which strengthen in their respective directions after controlling for grade, gender, school, and underrepresented minority (all p<0.001; Fig 2C). While continuous data were analyzed, visualization of the results are shown using quartiles for impulsivity to illustrate the magnitude of the stepwise effects (< = 28 [least impulsive]; 29–33, 34–37, and 38+ [most impulsive]), mindset (< = 55 [lowest mindset, referred to in the literature as “fixed” mindset], 56–60, 61–65, to 66+ [highest mindset, “growth” mindset]), and SSSE (< = 52, 53–67, 68–84, and 85+). Mean SSSE scores for students in the most impulsive quartile/highest mindset quartile (70.8±2.9; 95% CI = 65.2–76.5) were equivalent to students in the least impulsive/lowest mindset quartile (68.7±2.8 SE; 95% CI 63.3–74.1). Impulsivity and mindset’s relationship to STEM variables of interest (e.g., STEM interest, STEM skills) without adjusting for demographic factors is described in **Table 5**.

**Table 5:** Effect sizes of impulsivity and mindset on STEM metrics, ranked by impact. Impulsivity had large negative effects on sources of science self-efficacy (SSSE), STEM domain interest, and math interest. Mindset had a large positive effect on SSSE, with moderate-large effects on STEM domain interest, science interest, and interest in a STEM career.

Metrics	Marginal Mean±SE	95% CI	r	SS	Df, n	MS	F	Sig (p)	Effect Size (Partial η^2^)
**Impulsivity on:**									
Sources of Science Self-Efficacy	63.6 ± 1.31	61.1–66.2	-.43	179367.2	42, 1663	4270.6	9.980	0.000	0.206
Composite STEM Domains Score	91.3+1.83	87.7–94.9	-.32	52727.7	38, 566	1387.6	2.815	0.000	0.169
Mathematics Interest	20.6+0.68	19.2–21.9	-.29	8288.2	39, 642	212.518	3.100	0.000	0.167
Composite STEM Skills	13.8 ± 0.17	13.5–14.1	-.31	2348.3	42, 2064	55.9	6.464	0.000	0.118
Science Interest	22.7+0.63	21.5–23.9	-.24	4483.6	39, 647	115.0	1.970	0.001	0.112
Technology Interest	25.6+0.66	24.3–26.9	-.16	4309.6	38, 631	113.4	1.723	0.005	0.100
Interest in a STEM Career	23.9+0.7	22.5–25.3	-.18	4204.6	39, 630	107.8	1.537	0.022	0.092
Engineering Interest	21.9+0.71	20.5–23.3	-.19	3978.0	39, 624	102.0	1.412	0.053	0.086
**Mindset on:**									
Sources of Science Self-Efficacy	64.7+1.43	61.9–67.5	.41	166697.6	43, 1580	3876.7	9.241	0.000	0.206
Composite STEM Domains Score	96.5+1.89	92.8–100.2	.25	34907.6	36, 472	969.7	1.885	0.002	0.135
Science Interest	24.9+0.62	23.7–26.1	.23	4428.4	36, 530	123.0	2.082	0.000	0.132
Interest in a STEM Career	24.8+0.69	23.4–26.1	.17	4645.8	36, 518	129.049	1.844	0.003	0.121
Technology Interest	26.1+0.65	24.8–27.4	.18	3785.2	36, 520	105.1	1.668	0.010	0.111
Composite STEM Skills	14.4+0.2	14–14.8	.22	1275.5	43, 1750	29.7	3.136	0.000	0.073
Engineering Interest	23.9+0.72	22.5–25.3	.11	3625.7	36, 513	100.7	1.314	0.109	0.090
Mathematics Interest	21.9+0.72	20.4–23.3	.15	3297.3	36, 528	91.6	1.197	0.204	0.081

The GLM function within SPSS was used to analyze effect sizes for impulsivity and mindset (as continuous variables) on STEM metrics. Items are ranked by effect size (partial η^2^) using established benchmarks to define small (partial η^2^ = 0.01), medium (partial η^2^ = 0.06), and large (partial η^2^ = 0.14) effects [45, 46]. No variables were held constant when estimating effect size (e.g., gender, grade), which are modeled instead in Table 4. Pearson product moment correlations were generally negligible (r<0.30), with the exception of small correlations (r = |0.3–0.5|) observed between impulsivity and mindset on SSSE as well as between impulsivity and STEM domain interest (composite total) and STEM skills. A large effect of composite STEM skills was observed on SSSE (*F*(16,1889) = 26.37, p<0.001, partial η^2^ = 0.184, 95% CI = 57.1–63.6, r = 0.41).

### Impulsivity was negatively associated with math and science interest; higher mindset opposed impulsivity’s effect on science interest

Math interest and science interest were modeled with linear models, using impulsivity and mindset as independent continuous variables with the addition of grade, gender, and school as covariates. Baseline variables for the model were established using grade 6, male gender, and average impulsivity and mindset scores. The average science interest score for 6th grade male students with average impulsivity and average mindset is 23.46 (i.e. baseline science interest). Every unit increase in impulsivity was associated with a 0.22 unit decrease in science interest (95% CI: -0.34 - -0.11; p<0.001) while other variables were held constant (i.e., gender, grade). In contrast, every unit increase in mindset was associated with a 0.15 unit increase in science interest (95% CI: 0.05–0.26; p<0.01). No significant effects were observed for gender, grade, or school on science interest models. For math interest, the intercept (i.e. baseline math interest) was estimated to be 25.45 (95% CI: 21.69–29.20; p<0.001). Every unit increase in impulsivity was associated with a 0.37 unit decrease in math interest on average (95% CI: -0.49 - -0.25; p<0.001) while other variables were held constant (i.e., gender, grade, school). Mindset did not oppose this decrease (p = 0.51). School and gender had no effect on math interest, though each increase in grade level was associated with a 1.22 unit decrease in math interest (95% CI: -2.11- -0.34; p<0.01).

### Conserved relationship between impulsivity, mindset, SSSE, and math interest

To understand how these relationships may translate into learning behaviors in the classroom, students were asked dichotomous questions about how they solve math problems and the pace of their learning. Specifically, two questions asked students about their procedures when solving math problems, one asked about learning pace, and one asked about behaviors when working in a group setting. The impact of impulsivity on learning behaviors was subsequently examined by logistic regression to account for gender, grade and school in response selection. Impulsivity significantly influenced the probability of students’ selected response across all four items (Table 6). Specifically, when “doing long calculations”, the odds of students “finding checking my work tiresome” increased 1.07 times the odds of “repeating all my steps and checking my work carefully” for every unit increase in impulsivity after adjusting for gender, grade, and school (95% CI: 1.05–1.10, p<0.001). Likewise, when solving math problems, the odds of students “seeing the solutions but then have to struggle to figure out the steps” increased 1.07 times the odds of “working your way to the solution one step at a time” for every unit increase in impulsivity (95% CI: 1.04–1.10, p<0.001). The odds of learning “in fits and starts” was 1.06 times the odds of learning “at a fairly regular pace” for every unit increase in impulsivity (95% CI: 1.04–1.09, p<0.001). Finally, when “in a study group working on difficult material”, the odds of “sitting back and listening” compared to the odds of “jumping in and contributing” increased 1.06 for every increase in impulsivity, after adjusting for gender, grade, and school (95% CI: 1.03–1.09, p<0.01). Predicted probabilities for each impulsivity quartile are described in Table 6 along with how predicted probabilities shift in the context of SSSE, mindset, and math interest. For example, a student in the highest impulsivity quartile yet lowest quartile of SSSE, mindset, and math interest has a 0.71 odds of solving math problems by “see[ing] the solutions but then hav[ing] to struggle to figure out the steps” whereas a student in the lowest impulsivity quartile, yet highest quartiles of SSSE, mindset, and math interest has a 0.08 predicted probability of selecting that answer option (compared to "work my way to the solutions one step at a time”).

**Table 6:** Impulsivity significantly influenced the predicted probabilities of students’ learning strategy. Logistic regression was implemented to calculate odds ratios and predicted probabilities after adjusting for grade, gender, and school.

**Outcome**		**Parameters**	**Odds Ratio Estimate**	**t**	***Significance*** ***(p)***	**Odds Ratio** **95% CI**	**Predicted Probability of Impulsivity Quartiles (95% CI)**
**When I am doing long calculations…**		Intercept	1.06	0.14	p = 0.89	0.48–2.33	Q1 = 0.31 (0.24–0.39)
		Impulsivity	1.07	5.49	p<0.001	1.05–1.10	Q2 = 0.40 (0.33–0.48)
		Grade	0.94	-0.07	p = 0.47	0.78–1.12	Q3 = 0.47 (0.40–0.55)
		Gender	1.09	0.57	p = 0.57	0.80–1.50	Q4 = 0.60 (0.53–0.67)
**When I solve math problems…**		Intercept	0.45	-1.88	p = 0.06	0.19–1.02	Q1 = 0.14 (0.09–0.20)
		Impulsivity	1.07	5.06	p<0.001	1.04–1.10	Q2 = 0.32 (0.26–0.40)
		Grade	1.07	0.66	p = 0.51	0.88–1.29	Q3 = 0.33 (0.26–0.41)
		Gender	0.78	-1.45	p = 0.09	0.56–1.09	Q4 = 0.41 (0.34–0.48)
**I learn…**		Intercept	0.92	-0.20	p = 0.84	0.42–2.05	Q1 = 0.35 (0.28–0.43)
		Impulsivity	1.06	4.83	p<0.04	1.04–1.10	Q2 = 0.47 (0.39–0.54)
		Grade	1.06	0.62	p = 0.54	0.89–1.26	Q3 = 0.47 (0.40–0.55)
		Gender	1.52	2.62	p<0.01	1.11–2.07	Q4 = 0.56 (0.49–0.56)
**In a study group working on difficult material…**		Intercept	1.28	0.63	p = 0.53	0.59–2.77	Q1 = 0.34 (0.27–0.42)
		Impulsivity	1.06	4.72	p<0.01	1.03–1.09	Q2 = 0.47 (0.40–0.55)
		Grade	0.96	-0.41	p = 0.68	0.81–1.14	Q3 = 0.42 (0.42–0.57)
		Gender	0.76	-1.76	p = 0.08	0.56–1.03	Q4 = 0.60 (0.54–0.67)

**Grouping Category**				**Predicted Probability (95% CI)**			
Impulsivity	Mindset	SSSE	Math Interest	**When I am doing long calculations**	**When I solve math problems**	**I learn…**	**In a study group…**
4 (High)	1 (Low)	1 (Low)	1 (Low)	0.71 (0.57–0.83)	0.71 (0.56–0.82)	0.69 (0.54–0.81)	0.75 (0.61–0.85)
1 (Low)	4 (High)	4 (High)	4 (High)	0.33 (0.22–0.47)	0.08 (0.04–0.16)	0.32 (0.21–0.45)	0.24 (0.15–0.36)
1 (Low)	1 (Low)	1 (Low)	1 (Low)	0.47 (0.26–0.69)	0.63 (0.39–0.83)	0.60 (0.38–0.79)	0.68 (0.46–0.84)
4 (High)	4 (High)	4 (High)	4 (High)	0.58 (0.40–0.74)	0.11 (0.05–0.22)	0.40 (0.25–0.58)	0.30 (0.17–0.47)

Variables were coded as follows: Gender (Male = 0, Female = 1); Grade (6–12); and School (1–6). When “doing long calculations”, the odds of students “finding checking my work tiresome” will increase 1.07 times the odds of “repeating all my steps and check my work carefully” for every unit increase in impulsivity after adjusting for gender, grade, and school (95% CI: 1.05–1.10, p<0.001). Likewise, when solving math problems, the odds of students “seeing the solutions but then have to struggle to figure out the steps” will increase 1.07 times the odds of “working your way to the solution one step at a time” for every unit increase in impulsivity (95% CI: 1.04–1.10, p<0.001). The odds of learning “in fits and starts” was 1.06 times the odds of learning “at a fairly regular pace” for every unit increase in impulsivity (95% CI: 1.04–1.09, p<0.001). Finally, when “in a study group working on difficult material”, the odds of “sitting back and listening” compared to the odds of “jumping in and contributing” increased 1.06 for every increase in impulsivity, after adjusting for gender, grade, and school (95% CI: 1.03–1.09, p<0.01). Predicted probabilities shift in the context of SSSE, mindset, and math interest. For example, a student in the highest impulsivity quartile yet lowest quartiles in SSSE, mindset (“fixed”), and math interest has a 0.71 odds of solving math problems by “see[ing] the solutions but then hav[ing] to struggle to figure out the steps” whereas a student in the lowest impulsivity quartile, yet highest quartiles of SSSE, mindset (“growth”), and math interest has a 0.08 predicted probability of selecting that answer option (compared to "work my way to the solutions one step at a time”). School had no impact on any of the models and is not shown due to space constraints.

**Fig 3:**
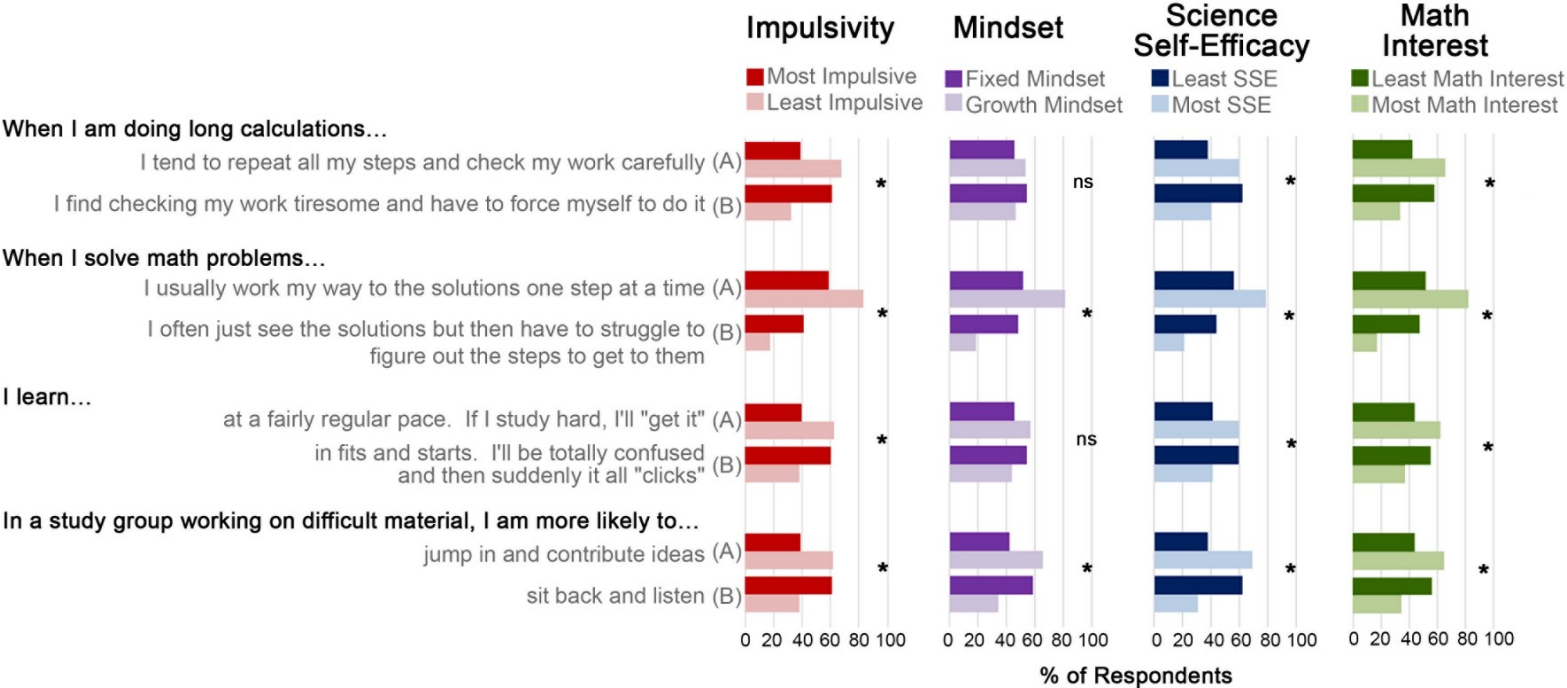
Learning behaviors are conserved between students in highest quartiles of impulsivity and lowest quartiles of SSSE and math interest. Each scale was binned into quartiles, with most impulsive students (dark red bars) reporting similar difficulties when solving math problems as students in lowest SSSE and math interest quartiles (darker bars, *p<0.002). Effects of impulsivity were analyzed by logistic regression (Table 6), with consistent results visualized using chi square tests of all four quartiles, though only highest/lowest quartile differences are shown here. Patterns were also consistent when examining students’ learning pace and behaviors when in a study group when working on difficult material (all p<0.002). Mindset quartiles displayed a similar pattern when solving math problems (p<0.001) and when working in a study group on difficult material (p<0.002), but not for long calculations (p = 0.37) or learning pace (p = 0.09). Logistic regression and chi square data are supported by independent samples t test results that found consistent differences in total scores depending on the answer option selected by students (Table 7).

When choice selection was visualized for all four questions (Fig 3), consistent patterns were observed where students in the highest impulsivity quartile were highly similar to students in the lowest SSSE and math interest quartiles (math quartiles: lowest = ≤16; 17–21; 22–29; 30+). With each stepwise increase in quartile, a proportion of students shifted to the other answer option. Mean scores of impulsivity, mindset, SSSE, and math interest were examined for each answer, with significantly different scores observed (Table 7). For example, “When [doing] long calculations…”, impulsivity scores were higher among students who responded that they ‘find checking [their] work tiresome and have to force [themselves] to do it’ (mean = 34.6) versus students who ‘tend to repeat all steps and check [their] work carefully (mean = 31.7; *t*(687) = -5.92, p<0.001).

**Table 7:** Relationship between mean scores for impulsivity, mindset, SSSE, and STEM domain interest based on learning strategy (choice selection) used to solve classroom behaviors.

	Impulsivity	Mindset	Sources of Science Self-Efficacy (SSSE)	Science Interest	Math Interest	Engineering Interest	Technology Interest	Interest in a STEM Career	Interest in STEM Domains (Cumulative Score)
**When I am doing long calculations…**	*t*(687) = -5.92, p<0.001^a^	*t*(563) = 1.65, p = 0.099	*t*(622) = 4.14, p<0.001^a^	*t*(1686) = 3.02, p<0.003 ^b^	*t*(1685) = 7.51, p<0.001^a^	*t*(1635) = 2.97, p = 0.004 ^b^	*t*(1662) = 0.98, p = 0.330	*t*(1664) = 6.11, p<0.001^a^	*t*(1463) = 5.29, p<0.001^a^
I tend to repeat all my steps and check my work carefully (A)	31.7, 6.3, n = 373	60.8, 6.8, n = 294	72.7, 21.4, n = 324	24.8, 7.8, n = 889	23.3, 8.7, n = 893	23.3, 8.7, n = 856	25.6, 8.2, n = 881	25.2, 8, n = 880	97.4, 24.2, n = 774
I find checking my work tiresome and I have to force myself to do it (B)	34.6, 6.5, n = 316	59.9, 7.3, n = 271	65.2, 23.9, n = 300	23.6, 7.9, n = 799	20.2, 8.7, n = 794	22, 8.8, n = 781	25.2, 8.1, n = 783	22.7, 8.8, n = 786	90.8, 23.6, n = 699
**When I solve math problems…**	*t*(710) = -5.39, p<0.001^a^	*t*(583) = 5.07, p<0.001^a^	*t*(641) = 5.40, p<0.001^a^	*t*(1752) = 3.04, p<0.003 ^b^	*t*(1755) = 10.4, p<0.001 ^a^	*t*(1701) = 2.69, p<0.008 ^b^	*t*(1729) = 1.65, p = 0.10	*t*(1732) = 4.92, p<0.001 ^a^	*t*(969) = 6.46, p<0.001^a^
I usually work my way to the solutions one step at a time (A)	32.1, 6.4, n = 491	61.3, 6.9, n = 397	72.2, 21.9, n = 440	24.6, 7.9, n = 1194	23.3, 8.5, n = 1192	23, 8.8, n = 1149	25.6, 8.2, n = 1164	24.7, 8.4, n = 1174	96.8, 24.1, n = 1042
I often just see the solutions but then have to struggle to figure out the steps to get to them (B)	34.9, 6.3, n = 221	58.2, 6.9, n = 188	61.9, 23.7, n = 203	23.4, 7.8, n = 560	18.7, 8.7, n = 565	21.8, 8.8, n = 554	24.9, 8.1, n = 567	22.6, 8.5, n = 560	88.4, 23.4, n = 485
**I learn…**	*t*(691) = -4.82, p<0.001^a^	*t*(567) = 2.68, p<0.008 ^b^	*t*(589) = 3.73, p<0.001^a^	*t*(1699) = 2.51, p<0.02 ^c^	*t*(1700) = 5.18, p<0.001^a^	*t*(1649) = 3.43, p<0.001^a^	*t*(1672) = 1.47, p = 0.14	*t*(1676) = 3.45, p<0.001^a^	*t*(1481) = 4.20, p<0.001 ^a^
At a fairly regular pace. If I study hard, I’ll “get it” (A)	31.9, 6.2, n = 367	61.1, 7, n = 297	72.4, 21.1, n = 330	24.7, 7.6, n = 894	22.9, 8.8, n = 890	23.4, 8.6, n = 851	25.7, 8, n = 874	24.8, 8.3, n = 866	96.9, 23.4, n = 775
In fits and starts. I’ll be totally confused and then suddenly it all “clicks” (B)	34.2, 6.6, n = 326	59.5, 7.2, n = 272	65.7, 24, n = 295	23.8, 8.1, n = 807	20.8, 8.7, n = 812	21.9, 8.9, n = 800	25.1, 8.3, n = 800	23.4, 8.7, n = 812	91.6, 24.6, n = 708
**In a study group working on difficult material, I am more likely to…**	*t*(712) = -4.84, p<0.001 ^a^	*t*(581) = 4.01, p<0.001 ^a^	*t*(643) = 7.00, p<0.001 ^a^	*t*(1756) = 6.87, p<0.001 ^a^	*t*(1761) = 6.19, p<0.001 ^a^	*t*(1706) = 5.18, p<0.001 ^a^	*t*(1735) = 4.81, p<0.001 ^a^	*t*(1739) = 5.53, p<0.001 ^a^	*t*(1534) = 8.02, p<0.001 ^a^
Jump in and contribute ideas (A)	31.9, 6.4, n = 365	61.4, 7, n = 303	74.7, 22.3, n = 340	25.4, 7.7, n = 916	23, 8.8, n = 923	23.7, 8.6, n = 881	26.2, 7.9, n = 896	25.1, 8.4, n = 898	98.7, 23.5, n = 803
Sit back and listen (B)	34.2, 6.4, n = 349	59.1, 6.9, n = 280	62.5, 21.9, n = 305	22.9, 7.9, n = 842	20.4, 8.6, n = 840	21.5, 8.8, n = 827	24.4, 8.3, n = 841	22.9, 8.4, n = 843	89.0, 23.9, n = 733

Independent sample t tests were used to compare total scale scores for students selecting dichotomous answer options, applying Levene’s test for equality of variance when reporting test statistics. Results shown as Mean, SD, and sample size. Significant differences reported at the p<0.001 ^a^, p<0.01 ^b^, and p<0.05 ^c^ levels.

### Missing data comparisons

Patterns of missing data were analyzed by instrument completion status, student demographics, and survey time points. A total of 1403 students (56.7%) completed all four Survey 1 scales (impulsivity, mindset, SSSE, and STEM skills), 734 (29.6%) partially completed scales (i.e., attempted all four scales, but did not fully complete at least one scale), 291 (11.8%) skipping at least one scale in entirety, and 48 students (1.9%) did not respond to any questions on all four scales. Completion status did not differ for students completing Survey 1 versus matched surveys (p = .90). Completion status was not affected by gender (p = .21), though grade had a significant effect (p<0.001) where 6^th^ grade students were less likely to complete all scales (37.4%) compared to other grades (59.9–69.6%). Rather than partial completions, 6^th^ grade students skipped entire scales (31.6%) compared to older students (2.2%-10.3%), possibly due to lack of time. No differences existed in instrument scores between Survey 1, Survey 2, or matched surveys for any of the instruments except SSSE, which was higher among students with matched surveys (M = 68.9, SD = 23.0, n = 671) compared to Survey 1 alone (M = 66.6, SD = 22.4, n = 1228; p<0.05). Students with partial or skipped instruments were grouped for analyses, though only mindset showed a significant difference based on completion status (p<0.01), with higher mindset scores among students completing all scales (60.2, SD 7.3, n = 1403) than partial completions (M = 59.0, SD = 7.2, n = 358). GLM was used to determine if differences existed in scores by completion status and demographics. When examined by completion status, only STEM career interest scores differed, where higher scores were observed among students with partial completions on other scales (p<0.05).

## Discussion

The research presented above confirms the positive association and large effect size between science self-efficacy and STEM domain interest demonstrated by others [3, 5, 6]. It also confirms a positive association between higher mindset and self-beliefs towards STEM [48], which this study expands to include sources of science self-efficacy (large effect size), interest in all STEM domains (small to large effect size), interest in a STEM career (moderate-large effect size), and self-beliefs in STEM skills, such as using data and interpreting graphs (large effect size) among students in grades 6–12. Consistent with previous findings showing impulsivity affecting academic performance in the context of ADHD and self-discipline [20, 24, 29], this manuscript reports a negative relationship between impulsivity on most measures of STEM studied, including sources of science self-efficacy (large effect size), interest in all STEM domains (moderate-large effect size for all domains except engineering, p = 0.053), interest in a STEM career (moderate effect size), and STEM skills (moderate-large effect size). These findings suggest that impulsivity is likely influencing STEM learning outside the context of diagnosed and undiagnosed ADHD, which is estimated to have a prevalence within the U.S. school population of 5.9%-7.1% [30], though up to 11% per parent self-report [49]. The data presented here offer that students fall along a continuum of impulsivity scores, with each unit increase in impulsivity negatively influencing SSSE by 1.35 units, even after controlling for school, gender, grade, and underrepresented minority background across a large, three state sample of adolescents in grades 6–12 (Table 4). Thus, while some students may have diagnosed or undiagnosed ADHD, these data support a larger reach of impulsivity that may negatively impact STEM persistence, possibly by influencing students’ self-beliefs in their STEM abilities.

These results are not designed to be causal, but rather offer preliminary support for the combined impact that the degree of impulsivity and mindset play as significant behavioral correlates of science self-efficacy (Fig 2C) and STEM interest. For example, students in the most impulsive/highest mindset group had identical sources of science self-efficacy (SSSE) scores to students in the least impulsive/lowest mindset group. As impulsivity is thought to be a stable trait, whereas mindset can be changed, these findings suggest that mindset interventions may be beneficial for improving impulsive students’ self-efficacy for science. Growth mindset interventions, which emphasize recognition for effort rather than achievement, have been shown to improve learning and achievement [48, 50–52], particularly among groups underrepresented in STEM domains [40, 53–57]. This may be particularly important, since currently, no classroom strategies have sufficient evidence for supporting learning gains among ADHD students, even following medication to alleviate symptoms [58, 59]. This research suggests potential for mindset interventions in the classroom, especially for students with highest impulsivity, and with respect to science and math, most notably.

These findings are supported by data describing similar patterns for how most impulsive students solve math problems and engage in learning (Fig 3, Tables 6 and 7), which mirror patterns observed for students with least science self-efficacy and least math interest. These cross-sectional findings offer that impulsive students may struggle more when solving math problems or learning difficult material, which may negatively influence self-beliefs in their abilities, consistent with previous reports [3]. Impulsive students are not at an academic disadvantage, as their ability to perceive situations differently and learn at a different pace may be an asset in some situations, as early literature supports the notion that impulsivity can have functional or dysfunctional effects [60]. For example, Tymms and Merrell [20] offer that blurting out answers may be an overt sign of cognitive engagement, where impulsivity may serve a positive function. Our data show that “when in a study group working on difficult material”, impulsive students were more likely to “sit back and listen” than “jump in and contribute ideas”. While seemingly counterintuitive, this finding may stem from impulsive students’ altered self-beliefs in their abilities when working on challenging material. For example, when restricting analyses to only the most impulsive quartile, students who “jump in and contribute ideas” had significantly higher sources of science self-efficacy scores (p<0.02), mastery experience sub-scores (p<0.01), science interest scores (p<0.05), and reported greater self-beliefs in their ability to interpret graphs (p<0.05) than equally impulsive students who reported to “sit back and listen”. No differences were observed for math interest (p = 0.07) or mindset (p = 0.13) between these students. Thus, opportunities may exist for supporting impulsive students in STEM as they engage in difficult material or problem-based learning. For example, impulsive students in the classroom may experience greater challenges staying on task during longer projects, getting started on homework or a term project early, or consistently studying in advance for a test. As impulsive students have difficulty delaying gratification, classroom strategies that may be beneficial include shorter-term reward schedules (i.e., breaking down a unit project into smaller steps or assignments), frequent check-ins to identify learning gaps early, and positive feedback that encourages effort and hard work, particularly during practice-intensive units that focus on step-by-step problem solving. While impulsivity had a strong negative influence on STEM interest and beliefs, higher mindset opposed impulsivity’s effect only for science interest and science self-efficacy, not math interest (Table 4). As impulsivity and mindset had stronger effects on science self-efficacy than science interest, it is possible that math self-efficacy may be more sensitive to mindset than math interest.

Consistent with prior studies documenting a gender gap in STEM [48, 61–63], this study observed females had lower sources of science self-efficacy, which confirm results from Britner and Parajes [64] using the same scale. This effect was not related to impulsivity, as no difference in impulsivity was observed between genders. Mindset scores were higher among females (p<0.05, though the effect size was small). Mindset interventions may be particularly beneficial for students who express interest in STEM but lack the background content knowledge in a STEM domain, making the work more challenging, albeit surmountable. When not prepared for academic difficulties, students’ self-beliefs in their abilities may be challenged [53, 54] and reduce STEM interest and engagement [3]. Finally, consistent with prior findings [65], 9^th^ grade students had lower sources of science self-efficacy, interest in STEM domains, interest in a STEM career, and mindset, as well as a slight but significant increase in impulsivity when compared to students in other grades. Given that 9^th^ grade is the time when students are told that their grades are first starting to ‘count’ towards college, students may feel greater stress to succeed academically and may decline STEM electives, particularly if grades are low and/or a student feels behind compared to peers.

Important limitations of this work relate to its lack of causal design as well as caution in interpretations for grades 10–12. The modeling adjusted for school, gender, and treated grade as a continuous variable in the model, which supported associations found between impulsivity and mindset on SSSE. However, ANOVAs in Table 2 analyzed grade as a nominal variable and while 12^th^ grade students have low sources of science self-efficacy scores, the smaller sample size limits confidence in making interpretations related to effects of gender, mindset, impulsivity, or STEM interest. In addition, the cross-sectional design separated surveys across two time points to ease survey fatigue, which resulted in a lower sample size when comparing relationships with STEM domain interest. While significant, greatest confidence can be attributed to relationships between impulsivity, mindset, and sources of science self-efficacy, as these measures were completed within the same survey and were highly reproducible in every school site studied. While a tendency for impulsive students to not complete a questionnaire was expected, this was not the case, as only mindset scores differed between students who completed all instruments versus students with partially completed or completely skipped instruments. Instead, 6^th^ grade students had the greatest amount of skipped instruments, rather than partial completions, likely due to survey length and limited time. Finally, while the context of STEM was examined in the current study, it is unknown whether a similar relationship would be observed in non-STEM fields (e.g., humanities, music). For example, if students were measured in the context these other fields, such as when analyzing literature or learning music, it is possible that these relationships may persist and describe more about academic engagement independent of the subject matter.

## Conclusion

This study offers that impulsivity may influence learning behaviors, STEM interest, and self-beliefs regarding STEM across a wider spectrum of adolescents than previously considered. Based on the data, it is hypothesized that STEM persistence and attrition may be attributable to students’ underexplored behavioral characteristics (e.g., impulsivity and mindset) that impede or reinforce STEM learning consistent with government findings (2012) that also identified intellectual engagement, motivation, and identification with STEM pursuits as critical for persistence in STEM majors. These behavioral correlates, with impulsivity in particular, may deserve more consideration among faculty, STEM programs, as well as secondary and postsecondary institutions when supporting struggling students in STEM.
